# A Novel Modulation and Demodulation Method Based on Binary Frequency Shift Keying for Wireless Power and Data-Parallel Transmission

**DOI:** 10.3390/mi13091381

**Published:** 2022-08-24

**Authors:** Peizhou Liu, Tiande Gao, Ruixuan Zhao, Zhaoyong Mao, Quanzhe Zhu

**Affiliations:** 1 School of Marine Science and Technology, Northwestern Polytechnical University, Xi’an 710072, China; 2 Xi’an Precision Machinery Research Institute, Xi’an 710076, China; 3 Shaanxi Semiconductor Advanced Technology Center Co., Ltd., Xi’an 710077, China

**Keywords:** frequency shift keying, wireless power transfer, data

## Abstract

It is usually necessary but difficult to achieve reliable communication between the primary side and pick-up side in the wireless power transfer (WPT) system due to magnetic interferences. In this paper, a novel parallel transmission method for wireless power and data is proposed, which is based on the frequency shift keying (FSK) modulation and demodulation. The data are transmitted by changing the working frequency of the inverter and then demodulated based on the phase-locked loop (PLL) technology. In this way, the signal before the rectifier circuit for the data demodulation can overcome the influence of power transmission on the data transmission. Finally, a 426 W prototype has been built to verify the effectiveness of the proposed transmission method. The experimental results showed that when the output power changed from tens of watts to hundreds of watts, the data transmission was capable of achieving a stable transmission with a 10 kbps baud rate.

## 1. Introduction

WPT technology has been widely employed due to its advantages of contactless connection between power source and load. In the fields of consumer electronics, electric vehicles, underwater devices and biomedical implants, wireless charging is becoming an increasingly popular way of power supply [1,2,3,4,5,6]. In some application scenarios, additional communication is usually required for power transmissions. For example, the automatic-guided vehicle (AGV) needs to obtain navigation information while receiving power. The implanted medical devices not only need to receive energy but also need to interact with the external monitoring devices.

Generally, the conventional transmission method of power and data can be divided into two categories; one is setting an independent data transmission channel and the other is that the power and data transmission share one common channel. For the first approach, wireless data transmission technologies such as Bluetooth, Wi-Fi, and radio-frequency (RF) communication can be employed in WPT systems for achieving information exchange [7,8]. These methods can easily achieve high-speed communication, but extra antennas are needed. It is also a good choice to use independent coils for near-field couplings. Reasonable coil design can reduce the mutual interference between electric energy and data transmission channels to a certain extent [9]. Such methods require additional data transmission channels and generally have a relatively large scale. The transmission method of wireless power and data via one common channel does not need an additional data communication antenna. The inherent coupling coils for power transmissions are exploited as an antenna for data transmission, where the data are injected on the power carrier by inductance or capacitance [10,11,12]. Usually, the working frequency of the power transmission channel and the data transmission channel is different to reduce the mutual interference. Nevertheless, the power transmission channel is a frequency selective channel, and the transferred data will be weakened by the resonance network of the power transmission channel. In order to reduce the interferences from the power channel to the data channel, high-order filters are usually added to the data channel to reduce the interferences. However, with the increase of the power transmission, the data transmission channel is inevitably affected.

The communication technologies used in these existing systems include amplitude-shift keying (ASK) modulation, the phase-shift keying (PSK) modulation, and the frequency-shift keying (FSK) modulation. Although the method based on ASK is simple in modulation and demodulation, its anti-noise performance is relatively poor; especially when the power transmission is relatively large, this modulation method can hardly work stably [13]. The method based on PSK has a relatively better anti-noise performance, but the demodulation method is usually more complex [14]. Therefore, the binary frequency shift keying (2FSK) modulation and demodulation technology is widely applied because it has better anti-noise performance than ASK technology and a simpler demodulation method than PSK [15]. However, the technology based on 2FSK requires two different carriers to transmit information. In order to reduce the impact of power transmission, the carrier frequency of data transmission is usually high. As mentioned above, the signal with high carrier frequency will be seriously attenuated by the power transmission channel. It is a good choice to use two frequencies close to the resonance point of the power transmission channel for data transmission, which can avoid the attenuation of the data carrier by power transmission channel. However, when the interval between the two frequencies is relatively small, it is a very challenging thing to demodulate the data through the conventional method that uses a band-pass filter and envelope detector. In order to improve the signal-to-noise ratio, automatic gain control (AGC) technology is often used to automatically adjust the amplitude of the signal [16,17,18].

In this paper, a wireless power and data transmission system using 2FSK modulation is proposed. Data modulation is achieved by switching the frequency of the inverter. The frequency modulation signal with data information can be obtained by collecting the signal in front of the rectifier circuit. Different from the conventional demodulation methods, the frequency-tracking characteristics of the PLL are used to identify the carrier frequency. The method proposed in this paper does not need an additional modulation circuit, and the carrier frequency used for data communication is similar to the resonant frequency of the power transmission circuit, so the data transmission is not affected by the power transmission; at the same time, the communication performance is not affected by the increase of power transmission.

The organization of this paper is as follows. The power transfer analysis is given in Section 2. In Section 3, the method of data modulation and demodulation is introduced. The interference between power transmission and the data transmission channel is discussed in Section 4. In Section 5, a prototype is built to verify the performance of the proposed method. Finally, the conclusions are summarized in Section 6.

## 2. Power Transfer Analysis

**Figure 1:**
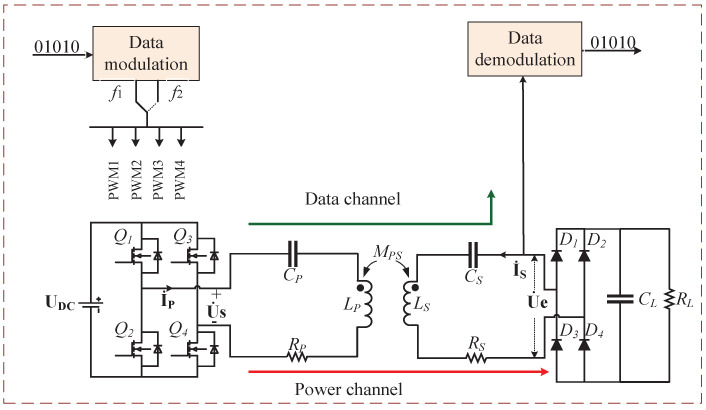
Schematic circuit of the proposed system.

There are various compensation topologies for efficient and stable power transmission. This paper emphasizes how to transmit data while transmitting electric energy. Hence, a series-series (S-S) compensated network is taken as an example because of its simple tuning. The schematic circuit of the proposed system is shown in Figure 1. The system consists of an inverter, two coupling coils, a rectifier, compensation networks, and demodulation circuits. The transmission of power and data is achieved through the same pair of magnetic coupling coils. The power transmission channel is exactly the same as the S-S compensated WPT system. The data transmission is achieved by the modulation and demodulation technology based on FSK; that is, the digital signal 0 or 1 is transmitted by changing the excitation frequency of the inverter. In order to reduce the influence of frequency change on power transmission efficiency, the default frequency of FSK modulation is the resonant frequency of the circuit, and the other frequency is slightly higher than the resonant frequency, which can ensure that the circuit always works in the soft switching state. Thanks to the function of the filter capacitor $C_{L}$, when the working frequency changes, a square wave can be obtained before the rectifier circuit. Data demodulation is achieved by sampling the voltage waveform in front of the rectifier circuit. In order to reduce the influence of the demodulation circuit as a load on the power transmission channel, the demodulation circuit is designed with high input impedance, which can be equivalent to an open circuit state for the power transmission channel.

**Figure 2:**
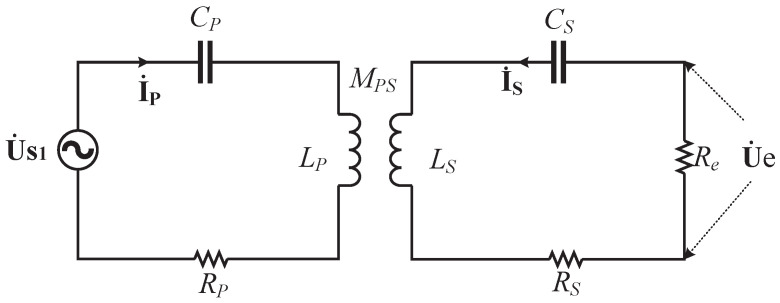
Equivalent circuit of the SS-compensated WPT system.

As shown in Figure 2, the equivalent circuit of the power transmission is completely consistent with the WPT circuit of S-S compensation, where $R_{p},R_{s}$ are the parasitic resistances of the primary and secondary coils; $L_{p}$ and $L_{s}$ are the self-inductances, respectively; $C_{p},C_{s}$ are the resonant capacitors connected in series with the two coils. $M_{PS}$ is the mutual inductance between the two coils; $R_{e}$ is the alternating current (AC) equivalent load at the rectifier circuit; $I_{p},I_{s},U_{s1},U_{e}$ are adopted to represent the phasor form of the corresponding variables. Based on the fundamental harmonic analysis (FHA), the following equation can be obtained

(1) $$\left\{\begin{array}{c} Z_{11}I_{p}+Z_{M}I_{s}=U_{s1}\\ Z_{M}I_{p}+Z_{22}I_{s}=U_{e} \end{array}\right.$$

where $U_{s1}$ represents the fundamental wave of $U_{s}$; $Z_{11}=R_{p}+j\omega L_{p}+\frac{1}{j\omega C_{p}}$; $Z_{M}=j\omega M_{ps}$; $Z_{22}=R_{s}+j\omega L_{s}+\frac{1}{j\omega C_{s}}$. Based on (1), the transmission ratio of voltage and current can be derived as follows

(2) $$\frac{I_{s}}{I_{p}}=\frac{-Z_{M}}{R_{e}+Z_{22}}$$

(3) $$\frac{U_{e}}{U_{s1}}=\frac{Z_{M}R_{e}}{Z_{11}\left(R_{e}+Z_{22}\right)-Z_{M}Z_{M}}$$

Then, the efficiency of power transmission can be expressed as

(4) $$\eta =\left|\frac{U_{e}I_{s}}{U_{s1}I_{p}}\right|=\left|\frac{-Z_{M}Z_{M}R_{e}}{\left(R_{e}+Z_{22}\right)\left(Z_{11}\left(R_{e}+Z_{22}\right)\right)-Z_{M}Z_{M}}\right|$$

When the circuit is fully resonant, that is, when the excitation frequency meets $\omega =1/\sqrt{L_{p}C_{p}}=1/\sqrt{L_{s}C_{s}}$, η can be expressed as

(5) $$\eta =\frac{\omega^{2}M_{PS}^{2}R_{e}}{\left(R_{e}+Z_{22}\right)\left(R_{p}\left(R_{e}+R_{s}\right)\right)+\omega^{2}M_{PS}^{2}}$$

Obviously, the frequency is an important factor affecting the efficiency of power transmission, so the parameter design for the data transmission channel needs to focus on the selection of the working frequency.

## 3. Data Transmission Method

In the proposed system, data transmission is achieved by the magnetic induction techniques. The communication antenna is also the coil for power transmission. The selection of data modulation method not only needs to consider the ability to resist the interference of power transmission but also needs to consider the impact on power transmission.

### 3.1. Data Modulation

In the proposed system, the binary data are directly modulated by changing the frequency of the inverter. It is assumed that when the inverter works at the resonant frequency, it represents the transmission symbol 1, and when the inverter deviates from the resonant frequency, it represents the transmission symbol 0.

**Figure 3:**
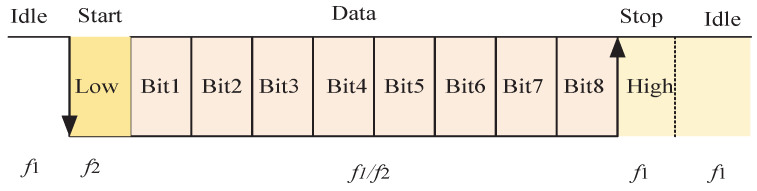
Schematic diagram of UART.

The universal asynchronous transceiver (UART) protocol is selected as the data communication protocol. As shown in Figure 3, in the idle state, the symbol 1 is always sent; that is, the inverter is always working in the resonant state. When it needs to send data, the inverter first sends a start bit 0 by working away from the resonant frequency operating point, then it sends 8 bits of data, and then it sends a stop bit after the data are sent, and finally, it returns to the idle state. Asynchronous communication recovers data by detecting the level of the data line. The accuracy of baud rate is an important factor affecting the reliability of communication. Baud rate deviation will increase the bit error rate and even lead to failure of normal communication. Therefore, the two frequencies must meet the following formula

(6) $$\frac{M}{f_{1}}=\frac{N}{f_{2}}$$

where M,N represent non-zero integers; $f_{1},f_{2}$ represent the two operating frequencies of the inverter. By sending the frequency $f_{1}$ for *M* cycles and the frequency $f_{2}$ for *N* cycles, it can be ensured that the length of time for sending high level or low level in the communication process is the same. It can be seen from (6) that a high baud rate can be achieved by increasing the frequency of the inverter or reducing the transmission time of a single symbol. In order to achieve the synchronous transmission of data and power, the selection of $f_{1}$ and $f_{2}$ also needs to consider the impact on the power transmission efficiency; that is, the frequency interval should not be too large. In addition, the phase continuity of carrier switching is also a factor that must be considered. Phase discontinuity will widen the spectrum of keying signals and produce parasitic radiation. The PWM signal controlling the inverter is usually generated by the timer of the micro control unit (MCU). The continuity of the phase can be guaranteed by setting the time of the internal timer as the cycle time of the carrier and generating the carrier as a complete integer cycle.

### 3.2. Data Demodulation

FSK can be demodulated by coherent demodulation or noncoherent demodulation. Coherent demodulation usually adopts two synchronous signals to be multiplied by the signal, respectively, and their outputs are added to the sampling decision circuit through the integrator, respectively, so as to output the required digital signal. In the WPT system, the generation of synchronous signal is a very challenging thing, so coherent demodulation is not applicable. Noncoherent demodulation is widely used because it does not require the generation of synchronous signals. The commonly used noncoherent demodulation method is filtering. First, the signal is passed through a group of band-pass filters; then, an envelope detector is used for envelope detection, and finally, the required digital signal is obtained through sampling decision [19]. When the two carrier frequencies of FSK are close, the band-pass filter needs a high order to suppress the out of band interference to the desired level. Therefore, when the power transmission increases, extracting two signals with relatively close frequencies requires increasing the scale and complexity of filter design.

**Figure 4:**
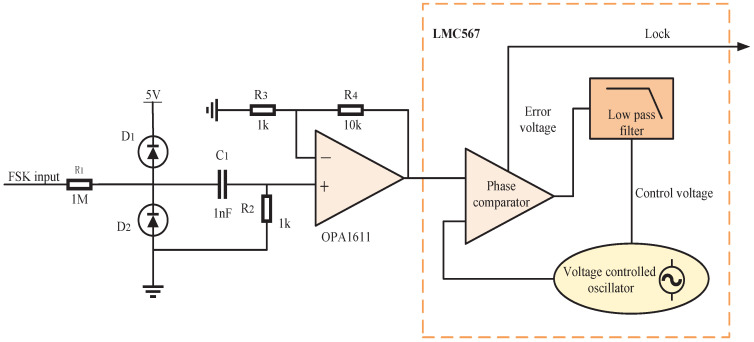
Schematic diagram of data demodulation.

In this paper, we use the PLL technology to demodulate the signal. The principle of demodulation is shown in Figure 4. The signal amplitude input to the operational amplifier is limited to 0∼5 V. The noninverting amplifier constructed by OPA1611 is used to amplify the signal by 11 times, which is mainly used to improve the signal-to-noise ratio of the input signal during low-power power transmission. In addition, the noninverting amplification circuit has a high input impedance, which can also reduce the impact of the data demodulation circuit as a load on the power transmission channel. The demodulation circuit consists of a phase detector, a loop filter, and a voltage-controlled oscillator. Through the control of loop filter bandwidth, the change of input signal frequency can be tracked, and a signal with the same frequency and phase as the input signal can be output through the negative feedback adjustment. In order to distinguish two different carrier frequencies, we set the tracking frequency center as the nonresonant frequency of power transmission circuit, and the capture band of the loop is less than the frequency difference between the two carriers. In this way, when the input signal frequency is consistent with the resonant frequency, the PLL will not be locked and output a high level. When the input signal frequency is the nonresonant frequency, the PLL can be locked normally and output a low level so as to achieve the demodulation of the FSK signal.

## 4. Crosstalk Analysis and Parameter Design

**Figure 5:**
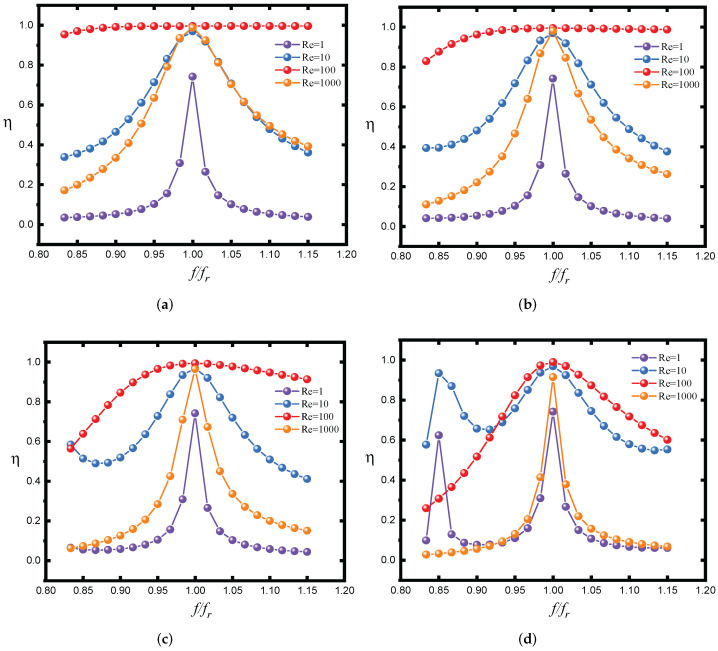
Variation of power transmission efficiency with working frequency ($L_{p}=L_{s}=52$ μH; $R_{p}=R_{s}=0.31\;\Omega$; $C_{p}=C_{s}=5.43$ nF; $f_{r}=300$ kHz). (**a**) k = 0.96. (**b**) k = 0.77. (**c**) k = 0.58. (**d**) k = 0.38.

In the proposed system, data and power are transmitted via one common channel; therefore, the following problems must be considered: firstly, the interferences from power transmission to data transmission and, secondly, the interferences from data transmission to power transmission. The method proposed in this paper modulates the data by directly changing the frequency of the inverter, and it demodulates the data by tracking the frequency of the square wave signal in front of the rectifier circuit. Therefore, it can be considered that the output power has no effect on data transmission. However, changing the frequency of the inverter will change the working state of the power transmission circuit, so it will affect the efficiency of power transmission. Figure 5 shows the change of power transmission efficiency with the working frequency under variable coupling coefficient k.

It can be seen from Figure 5 that when the coupling between the coils is relatively strong, the decline of power transmission efficiency is relatively flat when the working frequency deviates from the resonant frequency. The weaker the coupling between the coils, the more obvious the influence of frequency shift on power transmission efficiency. When the load resistance is 1 Ω, the power transmission efficiency is most sensitive to the frequency shift. A small load resistance will lead to a large reflection impedance. The primary side is close to open circuit and the secondary side is close to a short circuit. Similarly, when the load resistance is too large, the reflection impedance is very small, which will lead to the primary side close to short circuit and the secondary side close to open circuit. These two working states should be avoided when using S-S compensation topology.

Although increasing the carrier frequency can indeed improve the communication rate, the increase of the inverter switching frequency will not only increase the switching loss of the MOSFETs but also increase the parasitic resistance of the circuit due to the increase of the frequency. In view of the above reasons, we design the resonant frequency of the circuit as 300 kHz and choose another carrier frequency as 320 kHz. The ratio of the two frequencies is about 1.07. During communication, 15 cycles sent at 300 kHz represent the symbol 1, and 16 cycles sent at 320 kHz represent the symbol 0. This can ensure that each frequency can send a complete cycle in the communication process. In addition, sending multiple cycles to represent a symbol also leaves enough time for the acquisition and tracking of the demodulation circuit, which can reduce the bit error.

## 5. Experimental Verification and Discussion

**Figure 6:**
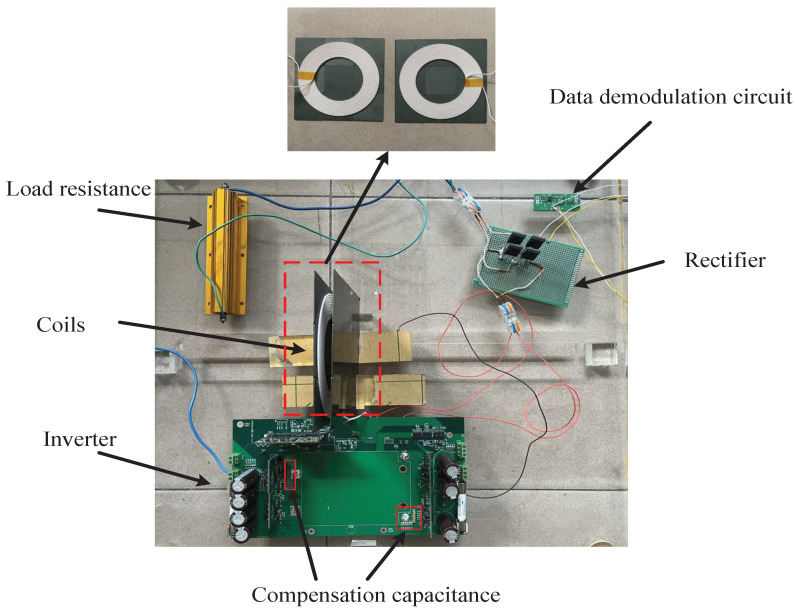
Picture of the prototype.

In order to validate the proposed method, a prototype is constructed as shown in Figure 6. Four SiC MOSFETs WM2M120080 and SiC didoes SA1D120020SC are used to build a full-bridge inverter and a full-wave rectifier, respectively. Litz wire with 0.1 mm × 100 strands is selected for coil processing in order to reduce the AC resistance. The mutual inductance between the two coils is obtained according to the in-phase and the opposing-phase connections of the coils [20], which is measured by an LCR-8205 impedance analyzer. The key parameters of the system are listed in Table 1.

**Figure 7:**
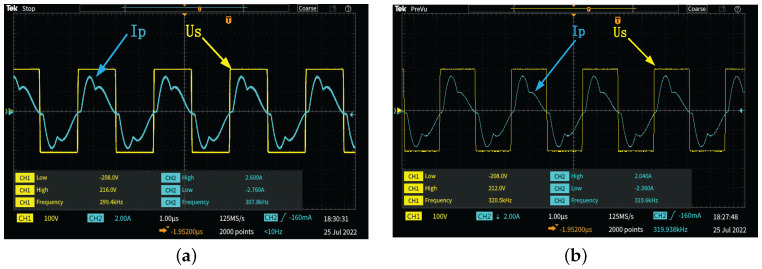
Voltage and current waveforms at inverter output under different frequencies. (**a**) f=300 kHz. (**b**) f=320 kHz.

When modulating the FSK signal, the working frequency of the inverter needs to work in the state of resonant frequency and nonresonant frequency. Figure 7 shows the waveform when the inverter operates at 300 kHz and 320 kHz. It can be observed that when the frequency of the inverter deviates from the resonance point, the phase difference between the current and the voltage does not increase significantly, which indicates that the resonant state of the circuit is not seriously affected when the frequency of the inverter deviates from the resonant point of the circuit. However, when the inverter works in the nonresonant state, the area surrounded by the current waveform and the coordinate axis is significantly reduced, which indicates that the output power is reduced.

**Figure 8:**
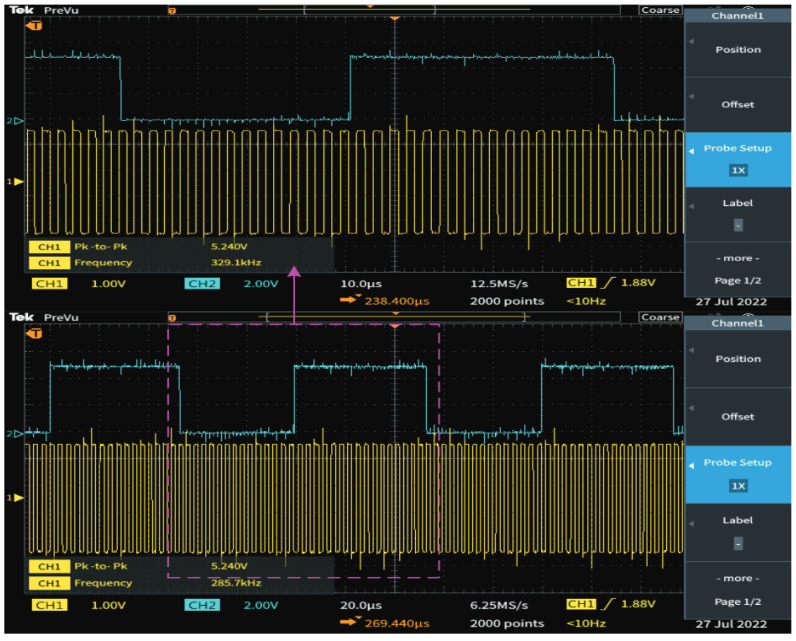
Voltage waveform of data demodulation.

The carrier of data modulation is 300 kHz and 320 kHz, and the bandwidth of the loop filter is set to 10 kHz, which can ensure that when the inverter works at these two frequencies, one frequency point can be locked normally, and the other frequency point cannot be locked so as to achieve data demodulation. In order to verify the effectiveness of the data demodulation circuit, the frequency of the inverter is switched every 100 uS; that is, 0 and 1 are sent periodically at a baud rate of 10 kbps. The input and output voltage waveform of the demodulation circuit are shown in Figure 8. The demodulation circuit can successfully recover the information modulated by the inverter. The lock and loss of PLL will take some time, so when modulating the signal, it needs to use multiple carrier cycles to represent a symbol bit. Compared with ASK, PSK and other demodulation methods, FSK needs to occupy a larger bandwidth.

**Figure 9:**
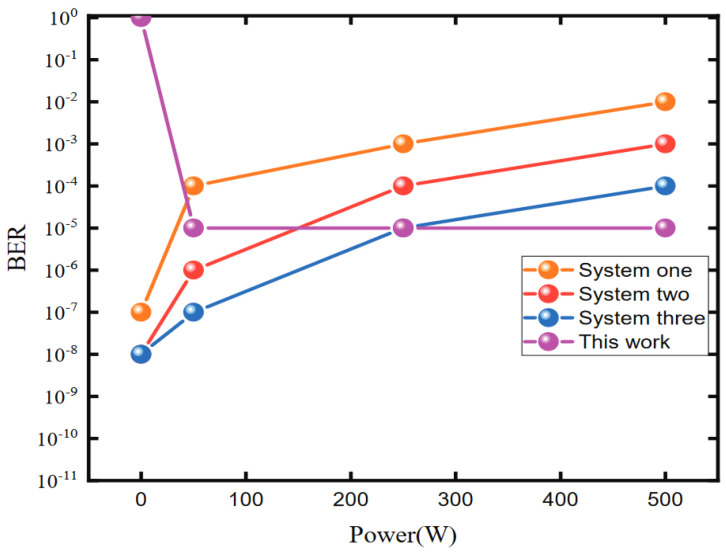
Variation of bit error rate with output power. System one corresponds to the system proposed in [13]; System two corresponds to the system proposed in [15]; System three corresponds to the system proposed in [21].

In the system where wireless power and data are transmitted together, the influence of electric power on data transmission is mainly reflected in the influence on communication error rate. In order to verify the influence of the output power on data transmission, the number 60 is sent continuously at the sending end according to UART protocol. The data are recorded in real time at the receiving end. By increasing the output power and analyzing the bit error rate of the data, the influence curve of output power on bit error rate can be obtained. Each group of experiments is tested for 10 min. At the same time, we simulate and compare the bit error rate of [13,15,21] under different output power for comparing the performance of the method proposed in this paper. The change curve of bit error rate with output power is shown in Figure 9. It can be seen that when the output power is small, the bit error rate of the proposed system is relatively large, and when the output power is about more than 10 W, the bit error rate level tends to stabilize. This is because at low output power, the signal received by the demodulation circuit end is relatively small, resulting in a low signal-to-noise ratio. When the output power increases, the signal at the data demodulation circuit end gradually increases and finally saturates. Therefore, the bit error rate tends to stabilize when the power increases to a certain extent. Compared with the proposed method, the bit error rate of the other three systems will increase with the increase of the output power. However, when there is no power transmission, the three systems can communicate normally, but the method proposed in this paper cannot work normally.

The other performance of the system presented in [13,15,21] is compared and tabulated in Table 2. The maximum communication rate is evaluated when the system can communicate normally at the output power. The communication rate of the system in [15] can reach 119 kbps. The output power of the systems in [15] is relatively small. The data carrier frequency of most systems is at the MHz level. Compared with the reported systems, the system proposed in this paper does not need an additional modulation circuit; hence, the circuit structure is simpler.

## 6. Conclusions

This paper proposed a power and data transmission method via one common channel based on FSK. Unlike the conventional wireless power and data transmission system, which uses an additional coupling circuit to achieve data modulation and a high-frequency carrier to reduce the interference from power transmission, the method proposed in this paper modulates the information by changing the frequency of the inverter in a small range, and it achieves data demodulation by using the frequency-tracking characteristics of the PLL. In addition, directly using the square wave signal in front of the rectifier circuit for data demodulation can make the data transmission unaffected by the increase of transmitted power. It has been verified experimentally that the proposed method works effectively and the data could be stably transmitted to the receiver at 10 kbps.

## Figures and Tables

**Table 1:** Parameters of the prototype.

Parameters	Definitions	Value	Unit
$M_{ps}$	Mutual inductance between the two coils	40.2	μH
$L_{p}$	Self-inductance of the transmitting coil	52.3	μH
$R_{p}$	Parasitic resistance of the transmitting coil	0.33	Ω
$C_{p}$	Compensation capacitance at transmitter	5.44	nF
$L_{s}$	Self-inductance of the receiving coil	52.2	μH
$R_{s}$	Parasitic resistance of the receiving coil	0.32	Ω
$C_{s}$	Compensation capacitance at receiver	5.43	nF
$R_{L}$	Load resistance	100	Ω
$U_{DC}$	Voltage of the DC power supply	200	V

**Table 2:** Performance of the existing system.

Design Metric	[13]	[15]	[21]	This Work
Modulation Method	ASK	ASK	ASK	FSK
Additional Modulation Circuit	YES	YES	YES	NO
Output Power	250 W	95.9 W	500 W	426 W
Power Frequency	39∼47 kHz	85 kHz	22.4 kHz	300 kHz/320 kHz
Data Carrier	1.5 MHz	850 kHz	1.67 MHz	300 kHz/320 kHz
Data Rate	19.2 kbps	119 kbps	20 kbps	10 kbps
AGC	NO	NO	NO	NO
Occupied Bandwidth	38.4 kHz	338 kHz	40 kHz	40 kHz

## Data Availability

Not applicable.
